# Mapping of UK Biobank clinical codes: Challenges and possible solutions

**DOI:** 10.1371/journal.pone.0275816

**Published:** 2022-12-16

**Authors:** Oleg Stroganov, Alena Fedarovich, Emily Wong, Yulia Skovpen, Elena Pakhomova, Ivan Grishagin, Dzmitry Fedarovich, Tania Khasanova, David Merberg, Sándor Szalma, Julie Bryant

**Affiliations:** 1 Rancho BioSciences, LLC, San Diego, California, United States of America; 2 Takeda Development Center Americas, Inc., San Diego, California, United States of America; 3 Takeda Development Center Americas, Inc., Cambridge, Massachusetts, United States of America; Cardiff Metropolitan University, UNITED KINGDOM

## Abstract

**Objective:**

The UK Biobank provides a rich collection of longitudinal clinical data coming from different healthcare providers and sources in England, Wales, and Scotland. Although extremely valuable and available to a wide research community, the heterogeneous dataset contains inconsistent medical terminology that is either aligned to several ontologies within the same category or unprocessed. To make these data useful to a research community, data cleaning, curation, and standardization are needed. Significant efforts to perform data reformatting, mapping to any selected ontologies (such as SNOMED-CT) and harmonization are required from any data user to integrate UK Biobank hospital inpatient and self-reported data, data from various registers with primary care (GP) data. The integrated clinical data would provide a more comprehensive picture of one’s medical history.

**Materials and methods:**

We evaluated several approaches to map GP clinical Read codes to International Classification of Diseases (ICD) and Systematized Nomenclature of Medicine Clinical Terms (SNOMED CT) terminologies. The results were compared, mapping inconsistencies were flagged, a quality category was assigned to each mapping to evaluate overall mapping quality.

**Results:**

We propose a curation and data integration pipeline for harmonizing diagnosis. We also report challenges identified in mapping Read codes from UK Biobank GP tables to ICD and SNOMED CT.

**Discussion and conclusion:**

Some of the challenges–the lack of precise one-to-one mapping between ontologies or the need for additional ontology to fully map terms–are general reflecting trade-offs to be made at different steps. Other challenges are due to automatic mapping and can be overcome by leveraging existing mappings, supplemented with automated and manual curation.

## Introduction

UK Biobank is a biomedical database and research resource supported by the National Health Service (NHS). It has collected information from half a million United Kingdom (UK) participants, including phenotypic, genomic, and imaging data from direct assessments, verbal interviews, on-line questionnaires, and electronic health records (EHR). The data continue to grow as the collection of biomedical data continues to expand with new assessments and longitudinal follow-up.

Over 500,000 volunteers were recruited between 2006 and 2010 through Assessment Centers designed specifically for this purpose. Data collected at the baseline visit included participant’s demographics, health and lifestyle, hearing, and cognitive function. A range of physical measurements were performed. samples of blood, urine, and saliva were collected followed by some web-based questionnaires A repeat assessment of 20,000 participants was carried out between August 2012 and June 2013 [1]. Imaging data collection was performed at two Imaging Assessment Centers beginning in 2014. In addition to data collected from Assessment Center visits and via online questionnaires, the UK Biobank dataset contains hospital inpatient data (HESIN) obtained through external providers, and data on cancer incidence and cause of death from national cancer and death registries.

A new primary care dataset recorded by health professionals working at general practices (GP) was first released for ~ 45% of the cohort in September 2019, followed by several COVID-19-specific data releases starting in 2020. The GP dataset contains coded prescriptions and clinical event information. These use new data formats and dictionaries and require significant effort in ontology mapping, reformatting, and harmonization for integration with hospital-inpatient and self-reported data, and data preparation for downstream analyses.

UK Biobank data are globally accessible for public health research. Authorized researchers can access and download datasets from the database system. The UK Biobank data dictionary showcase [2] presents available data for health-related research in a comprehensive and concise way and is continually under development as new data on exposure and health outcomes are incorporated into the database. Individual participants’ data are provided in a data table format (csv or txt). Most categorical variables are coded. Variable-specific coding dictionaries are openly available from the UK Biobank website, or can be accessed via NHS Digital Technology Reference data Update Distribution (TRUD) subscriptions [3].

Diseases supported by treatment information, including operations/procedures and medications, are the key clinical metadata categories required to create various phenotypes of interest for further data analysis, eg, creating cohorts for a wide range of clinical investigations or efficient exploitation of genomics data which requires linkage to patient phenotype profiles [4–7]. The coding system used by UK Biobank for these categories can be improved by curation/normalizing to one ontology.

For example, the disease/diagnosis-related clinical HESIN data are coded according to the World Health Organization’s International Classification of Diseases and Related Health Problems (ICD), whereas operations and procedures are coded according to the Office of Population, Censuses and Surveys: Classification of Interventions and Procedures (OPCS). English and most Welsh hospital data collected by UK Biobank are coded in ICD10 and OPCS-4, but earlier Scottish data are coded in ICD9 and OPCS-3. Some Welsh records are coded in ICD9. Clinically modified versions of the ICD used in the United States (ICD9-CM and ICD10-CM) are not used in the UK Biobank [8]. Data on cancer incidence and cause of death obtained from national cancer and death registries, respectively, are provided as ICD9 and ICD10. Moreover, verbal interview self-reported cancer and non-cancer illness are not aligned to ICD or any other terminology classification or ontology, and instead are given as free text descriptions to UK Biobank numeric codes.

GP data consist of three data tables, gp_registration.txt, gp_scripts.txt, gp_clinical.txt [9], and are recorded using several different controlled clinical terminologies. The clinical events data table, gp_clinical.txt, contains date and clinical code including (1) Read version 2 (Read v2 or Read2) and (2) Clinical Terms Version 3 (CTV3 or Read3) for various primary care events, such as consultations, diagnoses, history, symptoms, procedures, laboratory tests, and administrative information. Both Read codes are coded thesauruses of clinical terms widely used in UK primary care since 1985, providing a standard vocabulary for clinicians to record patient findings and procedures. Since 2018, primary care practices in the UK are migrating to using the Systematized Nomenclature of Medicine Clinical Terms (SNOMED CT) exclusively [9, 10], rendering Read2 and Read3 codes obsolete. This transition makes curation of UK Biobank clinical data cross-mapping of current interest–in order to be aligned with HESIN and other data Read2 and Read3 codes needs to be mapped to other clinical terminology such as ICD10 or SNOMED. The integrated clinical data would provide a more comprehensive picture of individual subjects’ medical history.

Despite efforts by UK Biobank to clean data [8], minimal GP data cleaning has been undertaken [9] necessitating its further curation and harmonization. Different approaches to overcoming challenges related to UK Biobank phenotype data have been described, such as a semi-supervised approach for rapidly creating clinical biomarker phenotypes [11], mapping UK Biobank data to the OMOP Common Data Model (OMOP CDM) using custom ETL framework Delphyne [12], and creating machine learning phenotyping models for specific diseases [7]. Natural Language Processing pipelines were applied to extract information embedded in unstructured text from clinical notes [13, 14], brain scan reports [15]. A number of studies investigate UK biobank data on specific diseases and put forward algorithms for harmonizing information related to diabetes [16], treatment-resistant depression [17], stroke [15, 18], dementia [19].

Initial investigation by authors of this paper revealed a need for a comprehensive approach towards UK Biobank data curation, since naïve automated mapping could inflate disease prevalence by two orders of magnitude [20]. We have developed an approach for UK Biobank GP clinical data curation through a combination of automated and manual steps to clean up and convert terms from similar categories to a uniform ontology, keeping consistency across UK Biobank datasets, creating dictionaries, and mapping files. An internal “fuzzy” terminology mapping tool (Fuzzy, [21]) is used for automated mapping of diseases to select ontologies (ICD10-CM, ICD-O-3, SNOMED CT). To reveal and understand GP mapping issues and confirm the efficiency of our curation approach, we ran a pilot mapping on a subset of Read codes (n~1,300). Some of the challenges that we identified are the lack of precise one-to-one mapping between ontologies, the need for additional ontology to fully map terms–are general reflecting trade-offs to be made at different steps. Other challenges are due to automatic mapping and can be overcome by leveraging existing mappings, supplemented with automated and manual curation.

## Materials and methods

### UK Biobank general practitioner (GP) data in Read v2 and v3 codes

The data table gp_clinical.txt was downloaded from UK Biobank in September 2019. It contains 123.7 M rows with clinical event data for 230,106 participants and has 8 fields including “eid” (Participant identifier), “data_provider” (1 = England (Vision), 2 = Scotland, 3 = England (TPP), 4 = Wales), “event_dt” (Date clinical code was entered), “read_2”, and “read_3” with coded clinical events, and three free text “value” fields.

#### Mapping ontologies

To map GP clinical Read codes to diseases the following ontologies were used:

- ICD9—International Classification of Diseases, Ninth Revision
- ICD10—International Classification of Diseases, Tenth Revision
- ICD10-CM—International Classification of Diseases, Tenth Revision, Clinical Modification
- ICD-O-3—International Classification of Diseases for Oncology v.3
- SNOMED CT—International Systematized Nomenclature of Medicine Clinical Terms

ICD9 codes were converted to ICD10 via UK Biobank coding87.txt alignment to coding19.txt manually and using “icd9_icd10” lookups from all_lkps_maps_v3.xlsx [22].

ICD10-CM used by Fuzzy is based on the content from Centers for Disease Control and Prevention (CDC) https://www.cdc.gov/nchs/icd/icd10cm.htm.

ICD-O-3 was downloaded from WHO ontologies https://www.who.int/standards/classifications/other-classifications/international-classification-of-diseases-for-oncology

SNOMED CT used by Fuzzy is based on https://uts.nlm.nih.gov/uts/. SnomedCT_InternationalRF2_PRODUCTION_20210131T120000Z version was used.

#### Semi-automated Read codes to ICD9 and ICD10 mapping using UK Biobank and TRUD look-up files

Most Read codes were automatically mapped to ICD9 and ICD10 using:

- UK Biobank mapping tables from all_lkps_maps_v3.xlsx for Read2 and Read3 codes alignment to ICD9 and ICD10 provided in the Resource 592 “Clinical coding classification systems and maps” [22],
- original NHSD TRUD mappings Descrip.v3, Icd9.v3, Icd10.v3, Terms.v3, V2termv3.v3 [23] to map Read2 and Read3 to ICD9 and ICD10 and Read descriptions.

Read codes mapped to ICD9 only were re-mapped to ICD10 using UK Biobank coding87.txt and coding19.txt. In addition, to map Read codes related to disease category but for which no mapping was provided by UK Biobank or TRUD, we used UK Biobank coding19.txt to manually convert Read descriptions to ICD10. Mapping was performed by the best match of ICD9 description (coding87.txt) or Read description to ICD10 description (coding19.txt). Online information https://icd.codes/convert/icd9-to-icd10-cm, https://icdlist.com/icd-10, www.icd10data.com, was used to justify the proper match including synonyms review. One of the rules used was if Read description is a high level like A42z./Read2 “Viral meningitis NOS”, and ICD10 has a choice of “Unspecified”, we use specific term in this case “A879 Viral meningitis, unspecified” rather than “A87 Viral meningitis”.

#### Manual mapping of Read codes to ICD9/ICD10

To validate automated mapping using UK Biobank and TRUD look-up files and to overcome issues, we selected a random subset of 1,313 disease-related Read codes from the GP clinical dataset. Selection of the random subset is described in S1 File.

Fuzzy was used for manual curation using the trigram method [24, 25] to do a fuzzy search in a pre-indexed public ontology. Trigram method was chosen because it allows to pre-index ontology data, unlike other methods that compare fuzzy terms live [21]. Implementation details are provided in S1 File. For each mapped term, Fuzzy provides a similarity score. A higher value score translated into a higher probability that the manually curated term will match the term mapped automatically. We mapped codes from one ontology to another ontology using Fuzzy. This was manually annotated to ICD10 using descriptions, including the possibility of using ICD10-CM and ICD-O-3 codes when ICD10 coding is insufficient. The manual mapping of selected Read codes was performed using the best match of Read description to ICD10-CM and ICD-O-3 descriptions and online resources https://icdlist.com/icd-10, www.icd10data.com. All manual annotations and evaluations were performed by MD and PhD with experience in clinical data curation and years of biomedical research.

In addition to manual curation and automatic mapping, we explored the use of additional information provided by TRUD for the selected subset.

The Read code list was automatically mapped to TRUD ICD9/ICD10 codes with mapping status E, G, D, and A based on Resource 592 “Clinical coding classification systems and maps” [22]. Unreliable mapping categories such as “R = Requires checking” were excluded:

- E = Exact one-to-one mapping. There is an exact match between host and target codes, with no alternatives.
- G = Target concept more general. The mapping is correct, but Read coded concept is more detailed, with no alternatives.
- D|C|C = Default mapping | refine flag “Completely refined” | add_code_flag “Complete. No further codes need to be added.”
- A|C|C = Alternative mapping | refine flag “Completely refined” | add_code_flag “Complete. No further codes need to be added.”

### Statistical analysis

95% confidence intervals were estimated using Bayesian approach and flat priors assumption as described in [26].

## Results

### UK Biobank curation workflows

**Fig 1:**
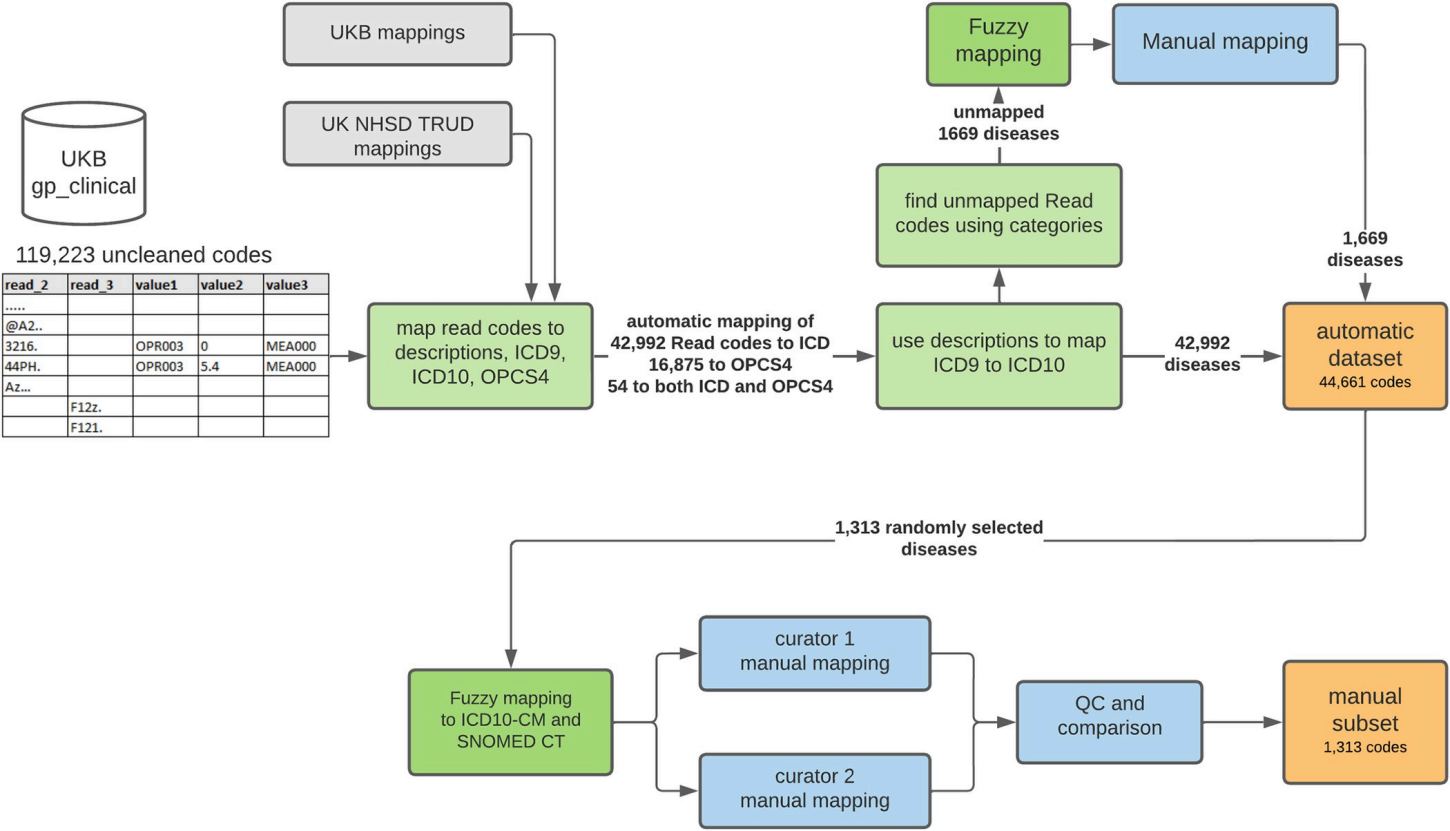
UK Biobank (UKB) GP clinical data curation—workflow for automatic and manual mapping of Read codes to ICD10, ICD10-CM, and SNOMED CT.

Workflow for large-scale automatic remapping of Read codes and for manual remapping is presented in Fig 1.

There are 38,228 unique Read2 and 80,995 Read3 codes in gp_clinical.txt, totaling 119,223 unique Read codes. These codes represent non-uniformed “clinical events” with a mix of several categories, eg, diseases, lab tests, occupations, etc. Read codes were first mapped to ICD9, ICD10, and OPCS4 using UK Biobank and UK NHSD TRUD mappings. As a result, 42,992 Read codes were mapped to ICD, and 16,875 Read codes to OPCS4, 54 Read3 codes had both ICD and OPCS4 mappings. ICD9 codes were further manually converted to ICD10. For QC and additional mapping, Read codes were converted to descriptions using coding files provided by UK Biobank and UK NHSD TRUD. We found that 54 Read3 codes mapped to both ICD and OPCS4 related to operational and therapeutic procedures. We also identified codes missing from UK Biobank and TRUD mappings. According to the Read hierarchy, some of these terms were classified as diseases (see S1 Table). Missing disease terms were mapped to ICD10 using Fuzzy. Terms with a similarity score below 80% were inspected and mapped manually. A threshold of 80% was based on evaluation of Fuzzy performance and was chosen because it provides a reasonable trade-off between accuracy and amount of manual curation [21].

Of 119,223 unique Read codes in the combined dataset, 44,661 were disease-related, which were mapped to ICD10, including 1,669 mapped manually: 14,098 (63 mapped manually) Read2 and 30,563 (1,606 mapped manually) Read3 codes. The remaining Read codes were considered unrelated to diseases and were ignored.

To evaluate the quality of automated mapping, 1,313 Read codes (Read2 and Read3) were selected at random. First, they were mapped to the codes from ontologies of interest (ICD10-CM and SNOMED CT) using Fuzzy matching method based on code descriptions. Manual mapping was then performed by two curators independently. The results were compared, inconsistencies were flagged, and final mapping was prepared (see S2 Table). Each manual mapping was assigned a quality category to evaluate overall mapping quality. In 76% cases terms found by Fuzzy were identical to manually mapped terms. For terms with similarity score below 1.0 the success rate was 58%.

Manual mapping of randomly selected 1,313 terms from Read codes to ICD10-CM codes revealed that only 796 (60.6%, 95% CI 58.0–63.2%) can be mapped precisely. Investigation of remaining 39.4% codes revealed that 439 (33.4%, 95% CI 30.9–36.0%) were mapped imprecisely, and 78 (5.9%, 95% CI 4.8–7.3%) were cases when one Read code was mapped to a multiple ICD10-CM codes. Full breakdown of mapping quality issues is provided in Table 1.

**Table 1:** Overall quality of mapping Read codes to ICD10-CM codes for a subset of 1,313 randomly selected Read codes from GP clinical data.

Number of codes (%)	Issues with mapping quality	Category*
796 (60.6%)	Perfect match: Read code is perfectly matched to ICD10-CM code	No issue
413 (31.4%)	ICD10-CM ontology does not contain a specific code corresponding to a Read code. More general ICD10-CM term has to be used for mapping. (Fig 2B)	Imprecise
36 (2.7%)	Some Read codes describe a group of conditions which can be explained by combinations of several ICD10-CM codes. (Fig 2A)	Multiple
27 (2.0%)	Some Read codes refer to tumor morphology terms. Additional ontology which is specifically for oncology presentation (such as ICD-O-3) should be used to map Read code correctly.	Multiple
16 (1.2%)	There is no ICD10-CM code which would match the Read code precisely. Therefore, the closest ICD10-CM term was selected. (Fig 2C)	Imprecise
15 (1.1%)	Some Read codes refer to general conditions and were mapped to a block of ICD10-CM codes. (For example, Read code “[X]Diseases of inner ear” was mapped to ICD10-CM block “H80-H83 Diseases of inner ear.”)	Multiple
10 (0.7%)	Some Read codes refer to general conditions and were mapped to specific ICD10-CM codes with “unspecified” or “uncomplicated” qualifiers. (For example, Read code “Rubella” was mapped to ICD10-CM code “B069 Rubella without complication.”)	Imprecise

* “Multiple” refers to one-to-many mapping. “Imprecise” means that curators were unable to find precise one-to-one mapping.

**Fig 2:**
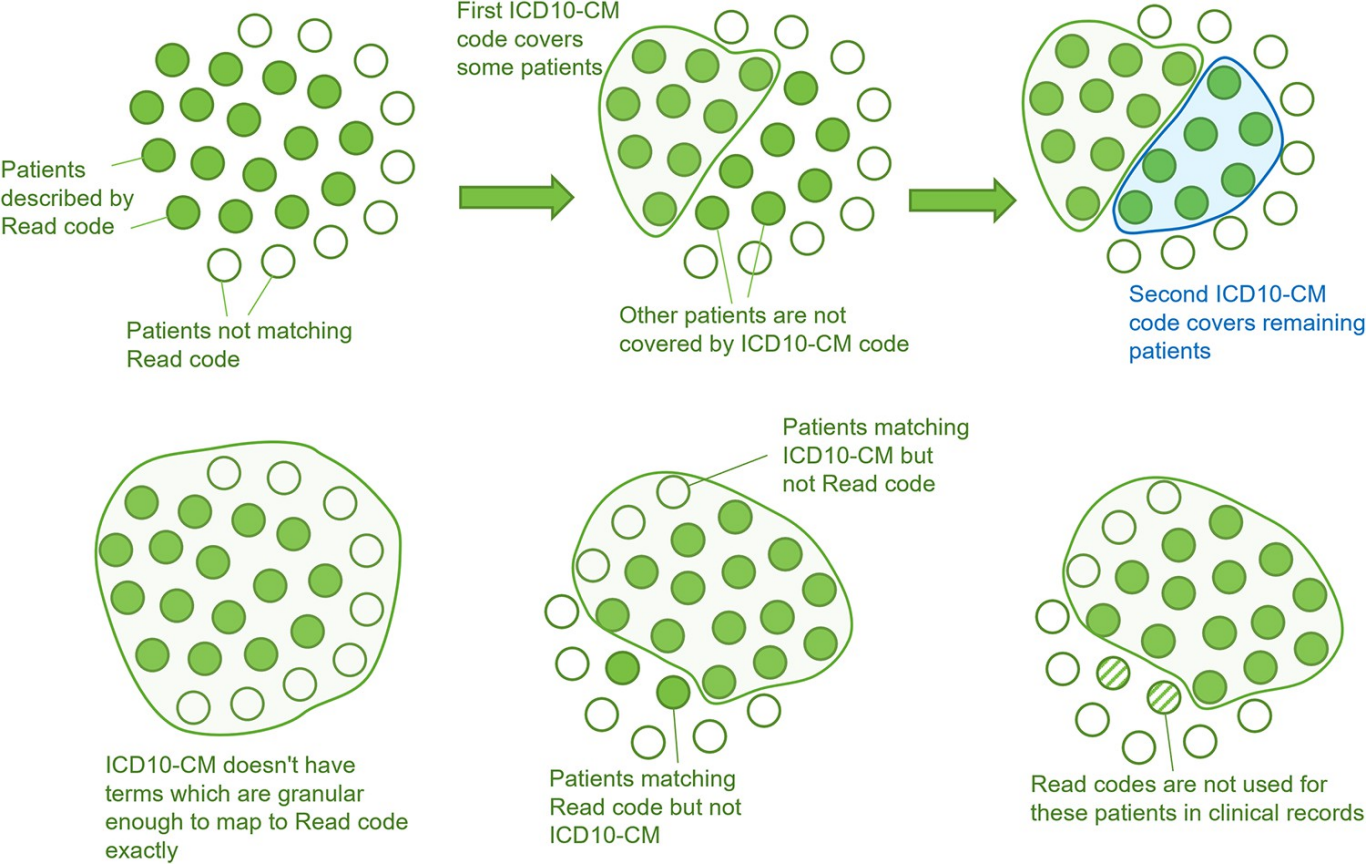
Illustration of term mapping issues due to mismatch between Read code and ICD10-CM ontology. Each circle represents a patient. Patients with Read code are represented by closed green circles. A–one Read code is mapped to several ICD10-CM codes. B–Read code is too specific for any ICD10-CM code, C–populations of patients described by ICD10-CM and Read codes do not overlap perfectly, D–hypothetical situation in which patients’ condition could be described by a Read code, but in clinical practice this code was not used.

Some issues are illustrated in Fig 2A–2C.

In 31.4% of cases, we were unable to find an exact ICD10-CM term that corresponded to a Read code, so mapping was done to a more general ICD10-CM term (see Table 2).

**Table 2:** Examples of mapping of Read codes to ICD10-CM codes. Mapping is performed manually or automatically using TRUD dictionary.

Read code, version and description*	ICD10-CM code and description mapped manually	ICD10-CM code and description mapped by TRUD
3/B3030 Malignant neoplasm of rib	C413 Malignant neoplasm of ribs, sternum and clavicle	
3/B7658 Benign neoplasm of perianal skin	D235 Other benign neoplasm of skin of trunk	
3/E2745 Jet lag–disorder	F518 Other sleep disorders not due to a substance or known physiological condition	
3/J07y2 Submandibular sialectasia	K118 Other diseases of salivary glands	
3/Xa5et Nizatidine allergy	Z888 Allergy status to other drugs, medicaments and biological substances	
3/F4A.. Keratitis &/or keratoconjunctivitis	H169 Unspecified keratitis, H16209 Unspecified keratoconjunctivitis, unspecified eye	
3/K1632 Diverticulitis of bladder	N323 Diverticulum of bladder, Q646 Congenital diverticulum of bladder	
2/J570. Anal and rectal polyp	K620 Anal polyp, K621 Rectal polyp	
3/66AJ0 Chronic hyperglycaemia	R739 Hyperglycemia, unspecified	
3/G2y.. Other specified hypertensive disease	I158 Other secondary hypertension	
3/A56.. Rubella	B069 Rubella without complication	
3/FyuQ. [X]Diseases of inner ear	H80-H83 Diseases of inner ear	
3/X78O6 Tumor of descending colon	D124 Benign neoplasm of descending colon, C186 Malignant neoplasm of descending colon	D489 Neoplasms of uncertain or unknown behavior, unspecified
3/Xa2Tq Dog bite of foot	W540XXA Bitten by dog, initial encounter	S913 Open wound of other parts of foot, S917 Multiple open wounds of ankle and foot
2/SN580 Egg allergy	Z91012 Allergy to eggs	T781 Other adverse food reactions, not elsewhere classified
2/A0762 Enteritis due to rotavirus	A080 Rotaviral enteritis	A080 Rotaviral enteritis, J108 Influenza with other manifestations, influenza virus identified, J118 Influenza with other manifestations, virus not identified

* 3/ or 2/ denotes v3 or v2 of the Read code.

### Automated mapping

The UK Biobank GP dataset contained 119,223 unique Read codes. To map this large number of Read codes to ICD10 or SNOMED codes, one would usually rely on publicly available mapping files and perform automated mapping. We explored an automated mapping workflow that utilized existing mapping files from UK Biobank and TRUD (Fig 1) and evaluated the performance by comparing mappings to those from manual curation for a subset of 1,313 randomly selected codes. We limited automated mapping to the first 4 letters of the ICD10 ontology and considered the mapping to be precise if those symbols matched the results of manual mapping.

As a basic approach for mapping UK Biobank codes, we used the mapping provided by TRUD and the lookup table provided by UK Biobank [3]. A comparison of automated mapping with manual mapping is given in Table 3.

**Table 3:** Comparison of the overall quality of mapping between Read codes and ICD10-CM codes for a subset of 1,313 randomly selected Read codes from GP clinical data.

Number of codes (%)	Quality of automated mapping when compared to manual mapping
418 (31.8%)	100% match to manually mapped
430 (32.7%)	4 letters of the ICD10 code match to manually mapped. Curators found more precise ICD10 terms, but automated mapping was correct
192 (14.6%)	3 letters of the ICD10 code match to manually mapped. Automated mapping was imprecise, but parent diagnosis term is most likely correct
123 (9.3%)	A Read code is mapped to several of the ICD10 codes by automated mapping. Some of the ICD10 codes were correctly mapped, while some were incorrectly mapped and should be excluded
90 (6.8%)	Automated mapping was close, but curators found better-fitting diagnosis
42 (3.2%)	Term in the publicly available mapping file was either missing or was incorrect and has to be replaced
18 (1.3%)	Read code refers to tumor morphology which should be mapped to ICD-O3

To address potential issues with one-to-many mappings, TRUD coding introduces a set of flags that provide additional information on the relationship between codes that are being matched. The *mapping status* flag denotes the nature of a mapping. For example, mapping status E means exact one-to-one mapping, G is used when target concept is more general, D and A mean “default” and “alternative” mapping (these flags are for situations in which Read code maps into a pair of target codes); R means “requires checking” against the default supplied in the table. The *Refine* flag specifies if target code is sufficiently detailed to be acceptable and the *add code* flag denotes whether the target system specifies that extra codes might be added to the target code. If a Read code is better explained by two or more ICD10 codes, these ICD10 codes are grouped into “blocks,” and alternative mappings within each block are given by the *element num* flag.

**Fig 3:**
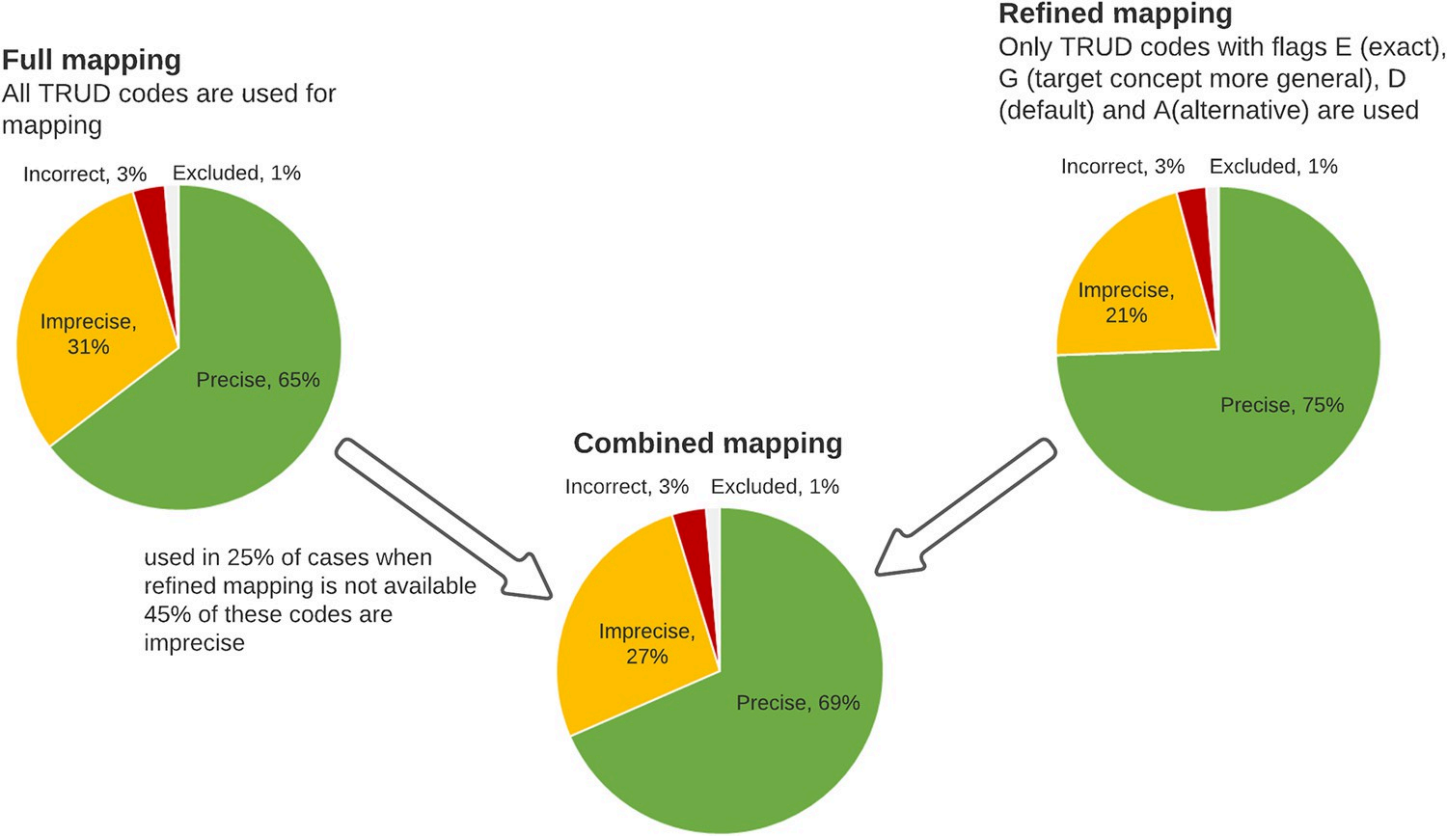
Workflow for using TRUD flags to refine automated mapping from Read codes to ICD10.

By restricting the list of mapped terms to terms with selected flags (eg, using only target codes with E, G, D, or A mapping status) it is possible to reduce the number of imprecisely mapped terms from 31% (95% CI 28.0–33.4%) for full mapping (where each target code is taken) to 21% (95% CI 19.0–24.1%) for refined mapping (where only selected Read codes are taken, Fig 3). However, 25% of codes would be excluded if we used refined mapping. One way to overcome this would be to use refined mapping when possible and use full mapping when refined mapping is not possible. However, this would still result in a high number (27%, 95% CI 24.5–29.3%) of imprecisely mapped terms.

### Mapping from Read codes to SNOMED

To evaluate the quality of Read-to-SNOMED CT mapping, we did a manual mapping between a previously selected subset of 1,313 Read codes and SNOMED CT. The algorithm was the same as the mapping between Read codes and ICD10 codes (see Fig 1): initial mapping was performed by Fuzzy, evaluated by two independent curators, results were compared, inconsistencies flagged, then the final mapping was prepared. We then compared results of manual mapping with mapping provided by SNOMED.

Out of 1,313 Read codes, there were 790 v3 Read codes, and 523 v2 Read codes.

The majority of v3 Read codes (97.8%) were found in SNOMED CT clinical terminology (only 18 of 790 v3 codes were missing). Read codes v2 are not present in SNOMED CT and therefore 523 were not mapped automatically. For these codes, only manual mapping was performed.

Of 790 Read v3 codes, 54% of manually mapped codes were identical to those provided by SNOMED CT. 42% of the Read codes were mapped by SNOMED CT to inactive terms. These were changed by a curator and more correct terms were found.

### Cross-mapping from Read codes to ICD with SNOMED CT

**Fig 4:**
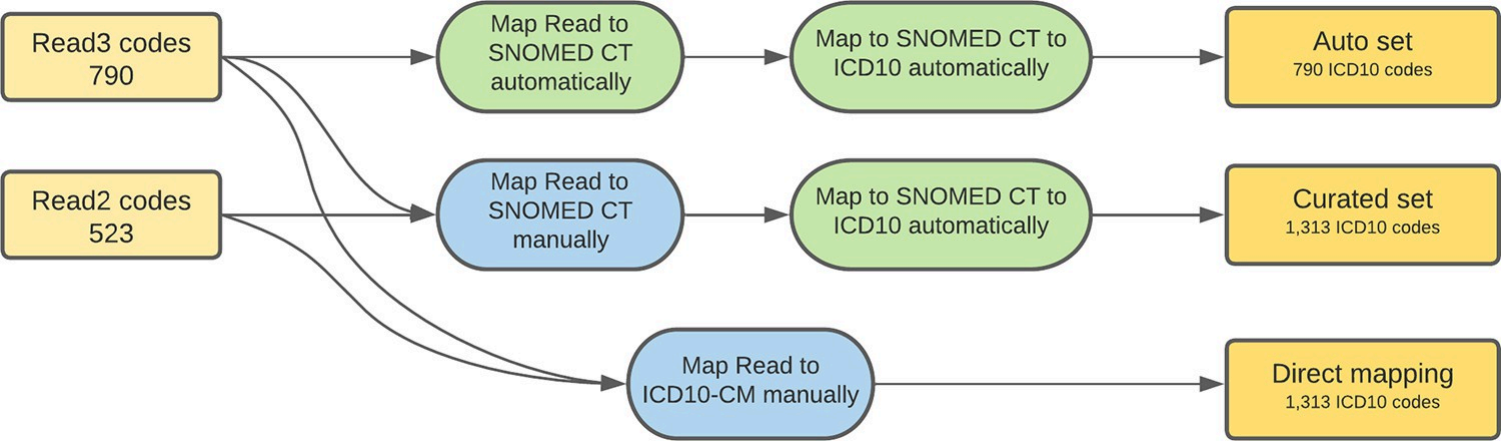
Workflow of a cross-mapping between Read codes and ICD10 codes using SNOMED CT.

To evaluate results of cross-mapping from Read codes to ICD10 codes, we used the mapping between Read codes and SNOMED CT, and fetched ICD10 information provided for the SNOMED CT terms (Fig 4).

We used SNOMED CT terms mapped manually or provided by SNOMED CT and compared results to a direct manual mapping from Read code to ICD10. The main issue with ICD10 codes generated from SNOMED CT codes mapped automatically was that ICD10 codes were missing for 43.4% of terms. The total amount of imprecise or missing codes was 65.9% (95% CI 62.6–69.2%) for fully automatic cross-mapping, compared to 48.2% (95% CI 45.5–50.9%) for cross-mapping using manually curated SNOMED CT codes. This is significantly worse than 31% (95% CI 28.0–33.4%) of imprecise and incorrect codes mapped using direct Read–to ICD10 described above.

## Discussion

### General challenges—Mismatches between disease ontologies

Challenges in linking the terms of one disease ontology to another that we highlighted are primarily due to mismatches between different disease ontologies. Most of the mapping issues were due to the absence of a precise equivalent for a Read code in the ICD10-CM ontology.

Frequently, Read codes contained specific localization of a condition that was absent from the ICD10-CM ontology, so general localization was chosen. In other cases, Read code terminology contained diseases or conditions caused by specific agents, for which only a general term was available in ICD10-CM ontology (see Table 2).

In 2.7% of cases a single Read code was mapped to a combination of ICD10-CM codes. Sometimes (for example, 3/F4A.. “Keratitis &/or keratoconjunctivitis”, or 2/J570 “Anal and rectal polyp”) individual conditions were explicitly mentioned, and for each individual condition we were able to find an ICD10-CM code. In other cases, a Read code described a condition without mentioning whether it was acquired or congenital, whereas the ICD10-CM ontology contained different terms, and so it was mapped to two ICD10-CM codes. Similarly, whenever tumor character was not explicitly described in a Read code, it was mapped to benign and malignant tumors in ICD10-CM ontology.

In 16 (1.2%) investigated Read codes we were unable to find the ICD10-CM or combination of ICD10-CM codes which could exactly fit. Sometimes, the main information about disease could exist in the ICD10-CM ontology, but the qualifiers (eg, congenital/acquired, malignant/benign) or location in the body disagree with the Read code description. These issues could be resolved by mapping to a higher level of ICD10-CM hierarchy. For example, there is no ICD10-CM code for 3/66AJ0 “Chronic hyperglycaemia.” One option is to map it to R73 “Elevated blood glucose level.” However, R73 contains several nested terms inconsistent with Read code because they might not be related to chronic hyperglycaemia, R7307 “Impaired fasting glucose,” or R7302 “Impaired glucose tolerance (oral),” leading us to map Read code to R739 “Hyperglycemia, unspecified” instead.

In some cases, it is impossible to map terms using just their descriptions, and one must take into account context as well as other terms in the ontology. For example, to provide a correct mapping of the Read code 3/G2y: “Other specified hypertensive disease,” one must know which hypertensive diseases are specified by other Read codes and exclude these diseases from the ICD10-CM codes. Based on that, the suitable ICD10-CM codes might include high-level terms such as I10-I16 “Hypertensive diseases,” I12 “Hypertensive chronic kidney disease,” I13 “Hypertensive heart and chronic kidney disease,” I15 “Secondary hypertension,” lower-level terms such as I151 “Hypertension secondary to other renal disorders,” I158 “Other secondary hypertension,” I159 “Secondary hypertension, unspecified,” or some combination thereof. Moreover, ambiguity in Read code description might lead to situations where the same patient could have been classified differently by different GPs, and the actual classification depends on the data collection practice.

In those and similar cases, there are two general approaches to mapping terms without knowing context:

- general ICD10-CM codes could be selected (eg, mapping of 3/G2y: “Other specified hypertensive disease” to the block I10-I16 “Hypertensive diseases” or mapping 3/FyuQ. “[X]Diseases of inner ear” to the block H80-H83 “Diseases of inner ear”),
- specific ICD10-CM codes for “unspecified” disease could be selected (mapping of 3/G2y: “Other specified hypertensive disease” to I158 “Other secondary hypertension,” or mapping of 3/A56.. Rubella to B069 “Rubella without complication”).

The rationale behind the second approach is that if the condition had a specific manifestation or type, it would have been recorded by a general physician using a specific Read code. However, this is an assumption: there is no guarantee that physicians used this logic to record the data, and it is practically impossible to get the reasoning behind selection of a particular code.

Our analysis shows that lossless mapping between Read code and ICD10-CM disease coding system is impossible due to mismatches in ontologies and unresolvable ambiguity in the way the Read code system was used by medical practitioners. This results in only 60% of codes being mapped without some loss of precision. The problem is exacerbated by using an automated approach.

### Automated mapping challenges

For 64.5% of the terms, the automated mapping produced perfect or nearly perfect results. Some of the errors were due to insufficiently precise or incorrect mapping as illustrated in Table 2.

**Fig 5:**
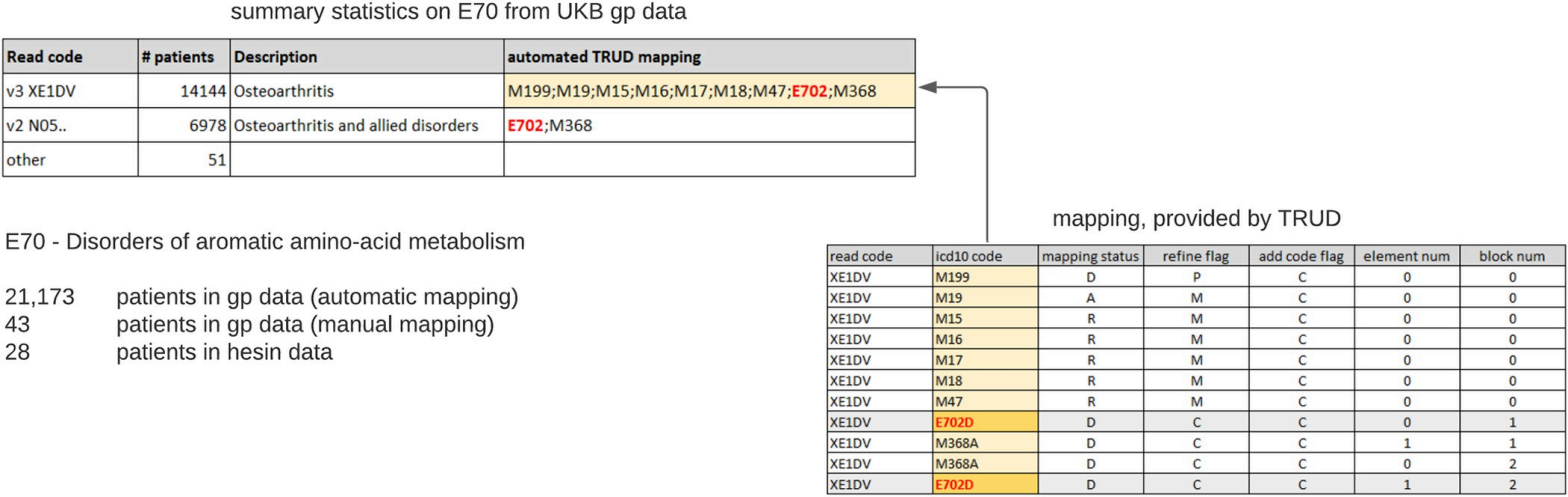
Automated mapping for ICD10 code E70 “Disorders of aromatic amino-acid metabolism”.

However, there were some errors that precluded the use of naïve automatic mapping for extraction of general statistics on diagnosis without further curation. In 9.3% of the cases, automated mapping returned several ICD10 codes for one Read code. In many cases these codes were incorrect, which resulted in highly inflated disease prevalence. One of the examples is illustrated in Fig 5. When we compared the number of patients with the ICD10 code E70 “Disorders of aromatic amino-acid metabolism” calculated using hospital inpatients (HESIN) and general physicians (GP) data, we found a huge discrepancy: only 28 cases were reported by HESIN data, whereas automatic mapping produced 21,173 GP patients. Further investigation revealed that two Read codes–v3 XE1DV “Osteoarthritis” and v2 NO5. “Osteoarthritis and allied disorders”–accounted for the majority of GP patients. For these Read codes, TRUD provided several matching ICD10 codes, and E702 was one of them. This leads to all patients with osteoarthritis being classified as having a disorder of aromatic amino-acid metabolism. When erroneous mappings were eliminated, the number of patients in GP data (51) became comparable with HESIN data (28) and manually curated GP data (43).

### Leveraging SNOMED CT to map Read codes to ICD10 codes

SNOMED CT was designed to replace Read codes [27]. Since 2018, primary care practices in the UK are migrating to SNOMED CT. Numerous SNOMED CT terms reference Read codes, and some of them reference ICD10 codes. We investigated whether we could improve the mappings of Read codes to ICD10 codes, by leveraging the Read-to-SNOMED CT and SNOMED CT-to-ICD10 mappings.

As with mapping between Read codes and ICD10 codes, differences in the data dictionary resulted in ambiguities. Sometimes these ambiguities could be resolved by using one-to-many mappings, but in many cases they might be impossible to make because no combination of target terms covers the source term exactly. If a certain degree of imprecision can be tolerated, there are two strategies of providing mappings: use either the smallest possible broad term, or the biggest possible narrow term (see Table 4).

**Table 4:** Examples of broad and narrow mapping between Read codes and SNOMED CT terms.

Read code	SNOMED CT	Description
3/F131.		Essential and other specified forms of tremor
broad	26079004	Tremor
narrow	609558009	Essential tremor (disorder)
3/N235.		Benign joint hypermobility
broad	85551004	Hypermobility syndrome
narrow	240262002	Localized benign joint hypermobility (disorder)
3/F1381		Dyskinesia: [orofacial] or [tardive]
broad	102449007	Tardive dyskinesia
narrow	118940003	Disorder of nervous system (disorder)
3/C040.		Hypothyroidism: [postsurgical] or [post ablative]
broad	40930008	Hypothyroidism (disorder)
narrow	27059002	Postoperative hypothyroidism

Which strategy to apply depends on the task being solved. Mapping to broad terms provides more robust phenotype prevalence data when disease records are summarized over the final terms. Mapping to narrow target terms misses other possible interpretations of Read codes (for example, a combination of several symptoms or states is mapped to one state). This makes prevalence data less reliable because it can inflate the number of patients for specific indications. However, mapping to narrow terms can be used to find additional information about populations that could be inaccessible when using general mapping.

## Conclusions

We revealed mapping issues of GP clinical codes due to inconsistencies in the use of ontologies believed to be compatible. Random sampling of Read codes showed that **33.4% (n = 439)** of mappings are imprecise. To overcome these issues, we suggested a few strategies, including using one-to-many, one-to-general, and one-to-specific mapping.

Each approach has advantages and disadvantages. A fully automated approach uses one-to-many mappings, leading to highly skewed disease prevalence data. Using cross mapping from Read codes to ICD10 through SNOMED CT dictionaries resulted in an even worse outcome.

Leveraging additional information about mapping flags from UK Biobank improves data quality. In our experience, the most robust approach would be mapping using a combination of automated and manual curation.

## Supporting information

S1 Table Read code hierarchy. Codes starting with capital letters A-Z are classified as diseases. Read2 and Read3 codes have similar hierarchy for the same Read code domains (0–9, capital letters A-Z).
(XLSX)

S2 Table Selected (n = 1313) Read code mappings to ICD10-CM performed using automated and manual approaches. A combination of ICD10-CM and ICD-O-3 codes is used for some neoplasms if ICD10-CM does not have a specific code.
(XLSX)

S1 File Supplementary methods. Selection of a random subset of Read codes. Trigram method.
(DOCX)
