# Correction: Mapping of UK Biobank clinical codes: Challenges and possible solutions

**DOI:** 10.1371/journal.pone.0316707

**Published:** 2024-12-27

**Authors:** Oleg Stroganov, Alena Fedarovich, Emily Wong, Yulia Skovpen, Elena Pakhomova, Ivan Grishagin, Dzmitry Fedarovich, Tania Khasanova, David Merberg, Sándor Szalma, Julie Bryant

S2 Table is uploaded incorrectly. Please view the correct S2 Table below.

## Supporting information

S2 Table (XLSX)
