# Supplementary material for: Mapping of UK Biobank clinical codes: Challenges and possible solutions
Source: PLoS One. 2022 Dec 16;17(12):e0275816. doi: 10.1371/journal.pone.0275816 (PMC9757572; doi:10.1371/journal.pone.0275816)
Supplement: S1 File — Selection of a random subset of Read codes. Trigram method. (DOCX) [file pone.0275816.s003.docx]

**S3 Methods. Supplementary methods.**

**Selection of a random subset of Read codes**

For a random Read code subset we used codes automatically mapped to ICD10. Codes beginning from A to Z (see Table S1) were selected of which categories T (Causes of injury and poisoning) and R ([D]Symptoms, signs and ill-defined conditions) were excluded. Selected Read2 and Read3 codes (n=38,980) were then separately shuffled using =rand() function in Excel followed by picking each 24th Read2 code using =MOD(ROW(),24) and each 33th Read3 code using =MOD(ROW(),33). We used this procedure to make sure relative proportion of Read2 and Read3 codes in our sample was close to the proportion in the bigger dataset.

**Trigram method**

Trigram method is part of n-gram family of methods (see e.g. https://www.w3.org/TR/ngram-spec/). The basic idea of the method is to generate an array of substrings of original string and the one we compare it with and then calculate the number of common substrings for both arrays, so e.g. for ‘leo’ the result will be {" l"," le","eo ",leo}.

While not as precise as e.g. double metaphone method, the trigram method allows pre-indexing (using both GIN [Generalized Inverted Index] or GiST [Generalized Inverted Seach Tree], see https://www.postgresql.org/docs/9.1/textsearch-indexes.html) , since we can pre-generate the list of substrings for each term. While indexing with trigrams takes some time, the actual similarity score calculation is fast enough to use in live applications. This means that once background indexing has completed, our implementation allows interaction with an ontology with sub-second speed.

Postgres implementation of trigram method is provided in /share/contrib/pg_trgm.sql file packaged with install. It provides us with similarity function and the % operator. The % operator allows for using a GIST/GIN index. There’s also a limit on similarity (0.3 by default) that can be set to speed up the search. Once all possible matches are found using % operator, found matches can be scored by similarity function (result will be between 0-1).
